# UVB-Stimulated TNFα Release from Human Melanocyte and Melanoma Cells Is Mediated by p38 MAPK

**DOI:** 10.3390/ijms140817029

**Published:** 2013-08-19

**Authors:** Visalini Muthusamy, Terrence J. Piva

**Affiliations:** School of Medical Sciences, RMIT University, PO Box 71, Bundoora VIC 3083, Australia

**Keywords:** UV, melanocytes, melanoma, TNFα, p38, JNK, NFκB, anisomycin

## Abstract

Ultraviolet (UV) radiation activates cell signaling pathways in melanocytes. As a result of altered signaling pathways and UV-induced cellular damage, melanocytes can undergo oncogenesis and develop into melanomas. In this study, we investigated the effect of UV-radiation on p38 MAPK (mitogen-activated protein kinase), JNK and NFκB pathways to determine which plays a major role in stimulating TNFα secretion in human HEM (melanocytes) and MM96L (melanoma) cells. MM96L cells exhibited 3.5-fold higher p38 activity than HEM cells at 5 min following UVA + B radiation and 1.6-fold higher JNK activity at 15–30 min following UVB+A radiation, while NFκB was minimally activated in both cells. Irradiated HEM cells had the greatest fold of TNFα secretion (UVB: 109-fold, UVA + B: 103-fold & UVB+A: 130-fold) when co-exposed to IL1α. The p38 inhibitor, SB202190, inhibited TNFα release by 93% from UVB-irradiated HEM cells. In the UVB-irradiated MM96L cells, both SB202190 and sulfasalazine (NFκB inhibitor) inhibited TNFα release by 52%. Although, anisomycin was a p38 MAPK activator, it inhibited TNFα release in UV-irradiated cells. This suggests that UV-mediated TNFα release may occur via different p38 pathway intermediates compared to those stimulated by anisomycin. As such, further studies into the functional role p38 MAPK plays in regulating TNFα release in UV-irradiated melanocyte-derived cells are warranted.

## 1. Introduction

The carcinogenic stimulus, ultraviolet (UV) radiation, can transform melanocytes into melanomas, which are an aggressive malignant skin cancer [1,2]. Both UVA and UVB radiation can penetrate into the epidermis and initiate molecular interactions leading to UV-induced responses. Some of these molecular interactions can give rise to genetic alteration, activation/suppression of cell signaling pathways, resulting in either the upregulation or downregulation of cytokine release. The molecular interactions of the B-Raf/extracellular-signal regulated kinase (ERK) pathway in melanoma cells have been widely investigated [1–3]. Unlike ERK, the other mitogen-activated protein kinase (MAPK); p38 MAPK and c-jun terminal kinase (JNK), and nuclear factor-κB (NFκB) pathways have not been frequently linked to melanoma incidences [1]. However, there is evidence to suggest that the inhibition/activation of these pathways together with chemotherapeutic agents can elicit cytotoxicity in melanoma cells [4–7]. These pathways have been suggested to also play a role in malignant transformation of melanocytes, although their anti-tumorigenic activities have also been reported [8–12]. Therefore, it is highly likely that besides the B-Raf/ERK pathway, the other MAPK pathways may on their own or in conjunction with ERK play a role in the development and progression of melanoma [3].

Denkert *et al.* [9] found that the p38 inhibitor, SB203580 caused a 60% reduction in the invasion of MeWo melanoma cells through a matrigel membrane. Estrada *et al.* [10] showed that the p38 MAPK/interleukin 8 (IL8) pathway was involved in melanoma cell migration and growth. Through the use of small interfering RNAs (siRNA), which reduced p38 MAPK activity, a decrease in IL8 expression was observed along with reduced migration of melanoma cells in a modified Boyden chamber. This inhibition was overcome by the addition of exogenous IL8, which confirms that this cytokine is downstream of the p38 MAPK pathway governing the migration of melanoma cells [10]. JNK inhibition was also shown to induce G_2_/M cycle arrest and render the melanoma cells susceptible to cell death [8]. Moreover, Ke *et al.* [13] found that the JNK pathway was involved in loss of cylindromatosis tumor suppressor function in melanoma cells thus enabling tumor growth and metastasis.

The NFκB pathway can be regulated by TNFα and other molecules resulting in changes to gene transcription [14]. McNulty *et al.* [15] when comparing Rel A expression observed that there were high levels in the nucleus of melanomas whereas it was mostly localized in the cytoplasm of benign naevus and only low levels were detected in normal melanocytes. In addition, Rel A was shown to play an important role in melanoma cell survival as antisense Rel A phosphorothioate oligonucleotides abrogated its protective effects [16]. Taken together, these findings suggest that the p38 MAPK, JNK and NFκB pathways are involved in both melanoma progression and metastasis.

Apart from changes to cell signaling activity, UV radiation can alter cytokine levels in melanocyte-derived cells [17]. Of interest is tumor necrosis factor-α (TNFα), a proinflammatory cytokine, which may be involved in anti- or pro-tumor activities in melanoma development [11,18]. Ivanov *et al.* [18] found that TNFα promoted cell survival of LU125 melanoma cells as the suppression of its expression led to UVC-induced (0.06 kJ/m^2^) cell death. In support of this finding, exogenous TNFα was found to inhibit apoptosis in melanoma cells with abrogated B-Raf signaling through the activation of the NFκB pathway [19]. Therefore, it is possible that TNFα and other molecules present in the tumor microenvironment may provide an added advantage for melanoma progression. However, TNFα has also been implicated in anti-tumor activities. It was used as an anti-vascular agent in melanoma cells where induction of TNFα in the tumor endothelium led to a breakdown of tumor vasculature and inhibition of tumor growth in mice [20]. As such, it will be crucial to delineate the pathways involved in mediating TNFα secretion from melanoma cells to selectively enhance or inhibit its levels.

In this study, we compared the effects of UV radiation on the activation of the p38, JNK and NFκB pathways, as well as TNFα secretion in primary human epidermal melanocytes (HEM) and a melanoma cell line (MM96L). The melanoma cell line was examined to see if the activity of these signaling pathways was altered during oncogenesis. Many studies have used UVC radiation to study cells signaling pathways, which are not physiologically relevant [18,21]. In this study, we used physiological doses, e.g., 1 MED (Minimal Erythemal Dose), to investigate the activation of cell signaling pathways following UV radiation. In addition, we also investigated UV-induced TNFα secretion from these melanocyte-derived cells using specific inhibitors like SB202190 (p38 MAPK inhibitor), SP600125 (JNK inhibitor) and sulfasalazine (NFκB inhibitor), in order to assist in determining which of these signaling pathways play a major role in this process.

## 2. Results

### 2.1. Effect of UV Radiation on the Viability of Melanocyte-Derived Cells

**Figure 1:**
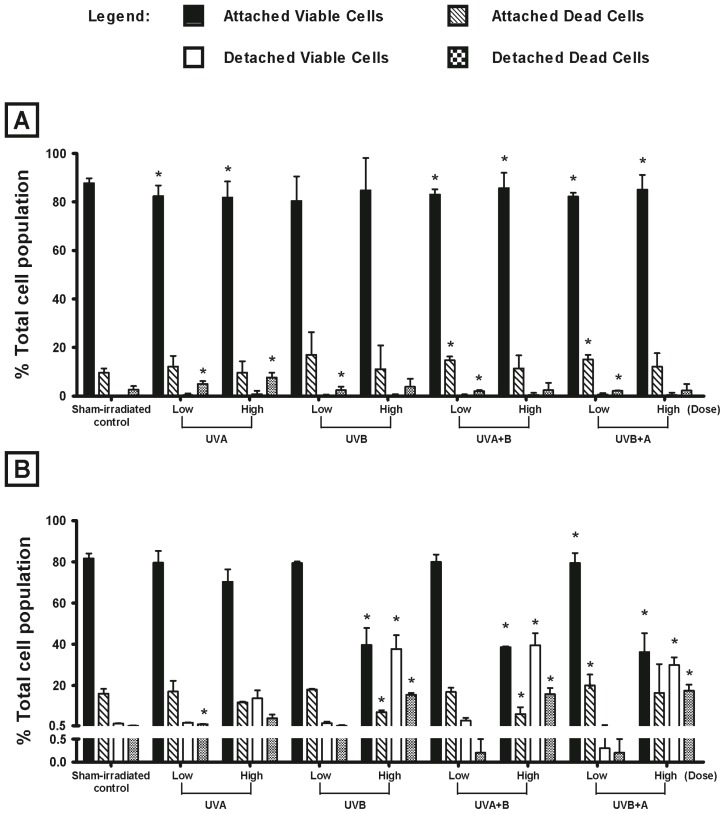
Effect of UV-irradiation on the viability of (**A**) human epidermal melanocytes (HEM) and (**B**) MM96L cell cultures at 24 h post-exposure. Cell viability was performed using trypan blue exclusion. Results expressed as the means ± SD from three independent experiments. Comparisons were made between sham-irradiated control and UV-irradiated cultures using Student’s paired *t*-test where significance was recorded as *p* ≤ 0.05 (*).

The effect of UV radiation (UVA, UVB, UVA + B or UVB + A) on the viability of HEM and MM96L cells were measured 24 h post-irradiation using trypan blue exclusion (Figure 1). Cells were exposed to either 40 kJ/m^2^ UVA and/or 2 kJ/m^2^ UVB, which is equivalent to the UV component found in 1 MED [22]. These doses are referred to as high dose. The low UV doses used in this study were equivalent to that seen in 0.1 MED (4 kJ/m^2^ UVA and 0.2 kJ/m^2^ UVB) and are referred to as low dose. As it was not possible to expose the cells to a simultaneous dose of UVA and UVB radiation together, they were either exposed to UVA first (referred to here as UVA + B) or UVB first (UVB + A). The purpose of these combined exposures were to see if the first UV type had an effect on the second type.

In this study, HEM cells do not appear to be sensitive to UV radiation as they had a high percentage of attached viable cells after exposure to low dose UV radiation that was similar to that seen in sham-irradiated controls (sham-irradiated control: 88%, low dose UV radiation: 80%–83%) (Figure 1A). A similar result was seen when the cells were exposed to high dose UV radiation (sham-irradiated control: 88%, high dose UV radiation: 82%–86%) (Figure 1A). In MM96L cells, low UV doses induced less cell death than that seen at the high dose (Figure 1B). Cultures exposed to high dose UVA had a higher percentage of attached viable cells when compared to those exposed to the other UV types (sham-irradiated control: 82%, high dose UVA: 70%, UVB: 40%, UVA + B: 39% UVB + A: 36%). The UV types containing UVB radiation were shown to be cytotoxic to MM96L cells. Overall, it can be seen that MM96L cells were more sensitive to UV radiation than were HEM cells when they were exposed to high doses of UV radiation (Figure 1).

### 2.2. Effect of UV Radiation on the Activation of p38 MAPK, JNK and NFκB Pathways in Melanocyte-Derived Cells

The activation of the p38 MAPK, JNK and NFκB signaling pathways, was observed over the first 120 min following UV radiation in HEM and MM96L cells. These cells were exposed to either a low or a high dose of UVA, UVB, UVA + B or UVB + A radiation to observe the effect these doses and UV types have on activating the signaling pathways in cells that have survived and/or accumulated a certain degree of damage (Figure 1). Once irradiated, the cell cultures were left to incubate for various amounts of time and cell protein lysates were extracted at the end of these time points to perform western blots.

#### 2.2.1. p38 MAPK Pathway

**Figure 2:**
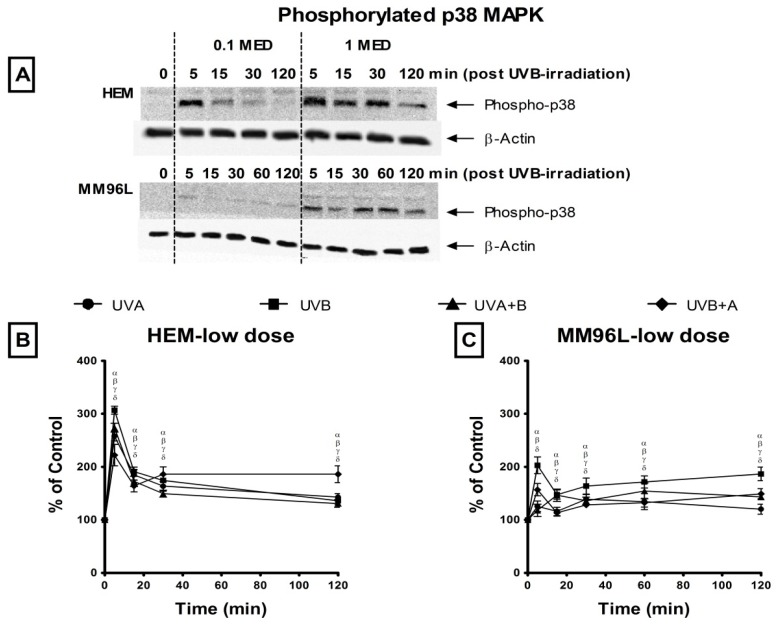

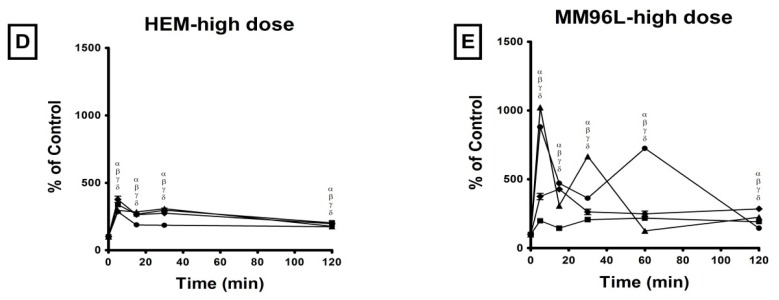
Effect of UV radiation on phospho-p38 expression in HEM and MM96L cells. (**A**) A representative western blot probed for phospho-p38 in HEM and MM96L cells post UVB-irradiation. Cell cultures were irradiated with either a (**B**,**C**) low (0.1 MED) or (**D**,**E**) high (1 MED) dose of UVA, UVB, UVA + B and UVB + A radiation. Cellular proteins were extracted at various time points (0–120 min) post-irradiation. In each lane 30 μg of cell lysate was added. Sham-irradiated control (0 min) = 100%. Results expressed as the means ± SD from three independent experiments. Comparisons were made between sham-irradiated controls and UV-irradiated cultures using Student’s paired *t*-test where significance was recorded as *p* ≤ 0.05 (α,β,γ,δ) [UVA (α); UVB (β); UVA + B (γ); UVB + A (δ)].

This pathway is activated following the phosphorylation of p38 MAPK [23]. Therefore, changes in phospho-p38 MAPK expression was measured in HEM and MM96L cells exposed to UV radiation and was expressed as a ratio of the UV-irradiated sample over the sham-irradiated control (0 min = 100%) (Figure 2A). In all cases, the level of phospho-p38 was first standardized against its loading control (β-actin) at each measured time point, and these values were used to calculate changes in expression as a result of UV exposure, as stated in the previous sentence.

In HEM cells, low dose UV radiation induced an immediate increase in phospho-p38 levels at 5 min post-irradiation with UVB stimulating the highest level (307% at 5 min) than the other UV types (UVA: 258%, UVA + B: 274%, UVB + A: 222%) (Figure 2B). A similar trend was observed after high dose UV radiation where phospho-p38 levels rose to 377% and 337% at 5 min post UVB + A and UVB radiation, respectively which was higher than that observed following UVA (286%) and UVA + B (296%) radiation (Figure 2D). In addition, high dose UVA radiation induced lower phospho-p38 levels than that of the other UV types as seen between 15 and 120 min post-irradiation.

In MM96L cells, low dose UV radiation induced less than a 2-fold increase in phospho-p38 levels (Figure 2C). In contrast, high dose UV radiation stimulated a greater increase in phospho-p38 levels, which rose to 880% and 1022% at 5 min following exposure to UVA and UVA + B radiation, respectively (Figure 2E). These levels remained elevated between 30 and 60 min post-irradiation. After UVB + A radiation, these levels peaked at 15 min (423%) while UVB radiation stimulated low phospho-p38 levels (<210%) during this 120 min period. Overall, low dose UV radiation reduced phosphorylation of p38 in MM96L cells compared to HEM cells, however high UV doses induced a dramatic increase in these levels in MM96L cells but not in HEM cells.

#### 2.2.2. JNK Pathway

**Figure 3:**
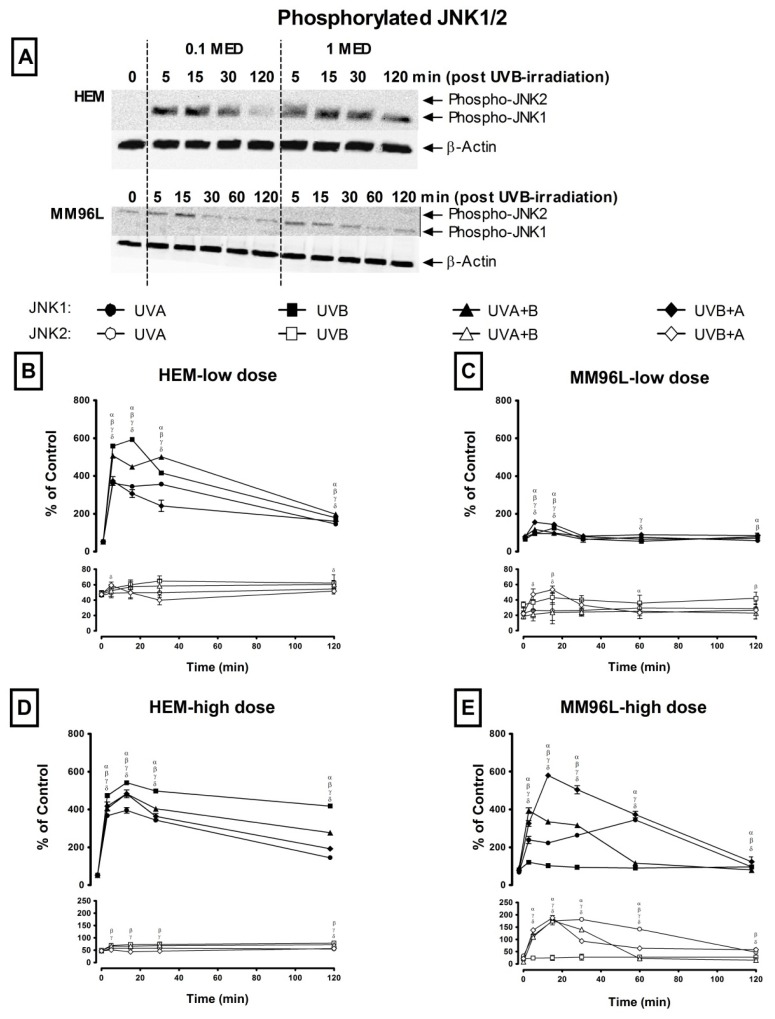
Effect of UV radiation on phospho-JNK1/2 expression in HEM and MM96L cells. (**A**) A representative western blot probed for phospho-JNK1/2 in HEM and MM96L cells post UVB-irradiation. Cell cultures were irradiated with either a (**B**,**C**) low (0.1 MED) or (**D**,**E**) high (1 MED) dose of UVA, UVB, UVA + B and UVB+A radiation. Cellular proteins were extracted at various time points (0–120 min) post-irradiation. In each lane 30 μg of cell lysate was added. Sham-irradiated control (0 min): phospho-JNK1 + -JNK2 = 100%. Results expressed as the means ± SD from three independent experiments. Comparisons were made between sham-irradiated controls and UV-irradiated cultures using Student’s paired *t*-test where significance was recorded as *p* ≤ 0.05 (α,β,γ,δ) [UVA (α); UVB (β); UVA + B (γ); UVB + A (δ)].

This pathway is activated when the JNK protein is phosphorylated by upstream activators [23]. As both the JNK1 and JNK2 isoforms are predominantly activated in skin cells [24], the expression of both phospho-JNK1 and -JNK2 was observed over 120 min post-exposure in HEM and MM96L cells (Figure 3A). Changes in the level of phospho-JNK1 or -JNK2 were expressed as a percentage of total phospho-JNK (JNK1 and JNK2) levels in sham-irradiated controls (0 min) (Figure 3A). In all cases, the levels of phospho-JNK1 and JNK2 were first standardized against their loading control (β-actin) at each measured time point. In the controls, the level of phospho-JNK1 + -JNK2 was added together and this total was expressed as 100%. The effect of UV exposure on the expression of each subunit at each time point was calculated as a ratio to that seen in the sham-irradiated controls as described above.

In HEM cells, low dose UV induced a 11-fold (~576%) increase in phospho-JNK1 levels between 5 and 15 min following UVB radiation while that after UVA + B radiation remained at 9-fold (~485%) until 30 min before declining to ~188% (4-fold) at 120 min post-irradiation (Figure 3B). Following UVA and UVB+A radiation, phospho-JNK levels increased by 7-fold (~360%) at 5 min post-irradiation. While these levels remained the same until 30 min following UVA radiation, that after UVB + A radiation began to decline to 160% at 120 min post-irradiation. After exposure to high dose UV, the pattern of phospho-JNK1 was similar in HEM cells where these levels peaked at 15 min post-irradiation, declining thereafter to varying degrees irrespective of the UV types used (Figure 3D). UVB radiation induced the highest levels of phospho-JNK1 (542% at 15 min) and remained high at 120 min (418%) post-irradiation while UVA induced the lowest levels (395% at 15 min and 145% at 120 min). UVA + B and UVB+A radiation induced a 9-fold (483%) increase in these levels, which fell to 277% (UVA + B) and 193% (UVB + A) at 120 min post-irradiation. Phospho-JNK2 levels were less than 2-fold in HEM cells exposed to either low or high dose UV radiation (Figure 3B,D).

In MM96L cells, phospho-JNK1 levels were highest (155% at 5 min) following low UVB+A radiation compared to the other UV types (UVA: 97% at 5 min, UVB: 125% at 15 min, UVA + B: 117% at 5 min) and these levels returned to control values (0 min = ~76%) at 30 min post-irradiation, irrespective of the UV type used (Figure 3C). Minimal phosphorylation of JNK2 was observed following exposure to low dose UV radiation (Figure 3C). In these cells, high dose UV radiation stimulated a rapid and sustained activation of phospho-JNK1 until 60 min post-irradiation except for those cells exposed to UVB radiation (Figure 3E). UVB + A radiation triggered a 7-fold (580% at 15 min) increase in phospho-JNK1 levels while there was only a 4 to 5-fold (~369%) increase following UVA (60 min) and UVA + B (5 min) radiation before it returned to baseline values (0 min = ~80%). Phospho-JNK1 levels were elevated less than 2-fold following UVB radiation, while phospho-JNK2 levels were also elevated (4–8 fold) after high dose UV-irradiation except for UVB-irradiated MM96L cells (Figure 3E). In general, HEM cells had higher phospho-JNK levels than did MM96L cells following low dose UV radiation but after high dose UV radiation, the former had lower levels except for those cells exposed to UVB radiation.

#### 2.2.3. NFκB Pathway

**Figure 4:**
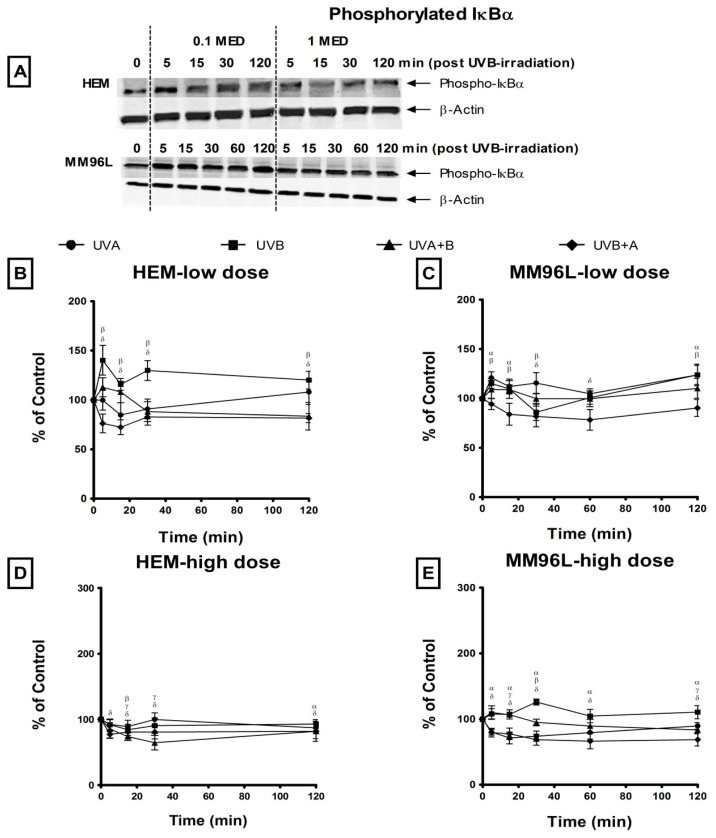
Effect of UV radiation on phospho-IκBα expression in HEK, HaCaT and Colo16 cells. (**A**) A representative western blot probed for phospho-IκBα in HEK and MM96L cells post UVB-irradiation. Cell cultures were irradiated with either a (**B**,C) low (0.1 MED) or (**D**,**E**) high (1 MED) dose of UVA, UVB, UVA + B and UVB+A radiation. Cellular proteins were extracted at various time points (0–120 min) post-irradiation. In each lane 30 μg of cell lysate was added. Sham-irradiated control (0 min) = 100%. Results expressed as the means ± SD from three independent experiments. Comparisons were made between sham-irradiated controls and UV-irradiated cultures using Student’s paired *t*-test where significance was recorded as *p* ≤ 0.05 (α,β,γ,δ) [UVA (α); UVB (β); UVA + B (γ); UVB + A (δ)].

The NFκB dimeric complex is freed when IκBα is phosphorylated and removed by the proteasome [14,25]. The addition of MG115 (proteasome inhibitor) prevents the degradation of phospho-IκBα thereby allowing for its accumulation within the cell [26]. As such, higher levels of phospho-IκBα would indicate an increase in the activation of NFκB (Figure 4A). Changes in the level of phospho-IκBα was expressed as a ratio of the UV-irradiated sample over its corresponding sham-irradiated control (0 min = 100%) (Figure 4A).

In HEM cells, following low dose UVB radiation, there was a slight increase in phospho-IκBα levels at 5 min (140%) post-irradiation while UVA and UVA + B radiation did not significantly after these levels throughout the 120 min time period (Figure 4B). In addition, phospho-IκBα levels fell below control levels (100% at 0 min) to ~78% from 5 to 120 min following UVB + A radiation. In these cells, high dose UV radiation did not stimulate an increase in phospho-IκBα levels as these levels were below control levels (≤100%) (Figure 4D). In MM96L cells, low dose UVA and UVB radiation induced a slight increase (<120%) in phospho-IκBα levels but these levels fell below control values (0 min = 100%) to 86% when cells were exposed to UVB+A radiation (Figure 4C). After high dose UV radiation, phospho-IκBα remained below control levels except for that following UVB radiation which peaked at 30 min (126%) post-irradiation (Figure 4E). In general, UV radiation induced little or no phosphorylation of IκBα in either HEM or MM96L cells.

### 2.3. Effect of UV Radiation and IL1α on TNFα Release in Melanocyte-Derived Cells

Cell signaling pathways are required to regulate the levels of cytokines present in the microenvironment of the skin in response to UV radiation. A range of cytokines including IL1, IL4, IL6, IL8, IL10, IL12, IL15 and TNFα are secreted by cells found in the epidermis and dermis [23]. Of these cytokines, IL1α and TNFα are considered to play an important role in UV-induced inflammatory and immunological responses [23,27–29]. Bashir *et al.* [30] found that IL1α (10 ng/mL) upregulated TNFα levels via increased gene transcription following UVB radiation in keratinocytes [30]. The production of IL1α by keratinocytes, fibroblasts and other cell types in the skin can act in a paracrine fashion to stimulate melanocyte cells. In sham- and UV-irradiated HEM cells, IL1α (10 ng/mL) did not significantly affect the activation of the JNK and NFκB pathways but induced a ~3-fold increase in phospho-p38 levels at 5–30 min post high dose UV-irradiation (results not shown). In MM96L cells, IL1α stimulation increased phospho-JNK1 and p38 levels by ~2-fold in the first 30 min of high dose UV-irradiation but no changes in phospho-IκBα levels were observed (results not shown). We then examined the effect exogenous IL1α had on TNFα secretion in cells exposed to 1 MED UV radiation as low dose UV radiation did not stimulate detectable amounts of secreted TNFα.

**Figure 5:**
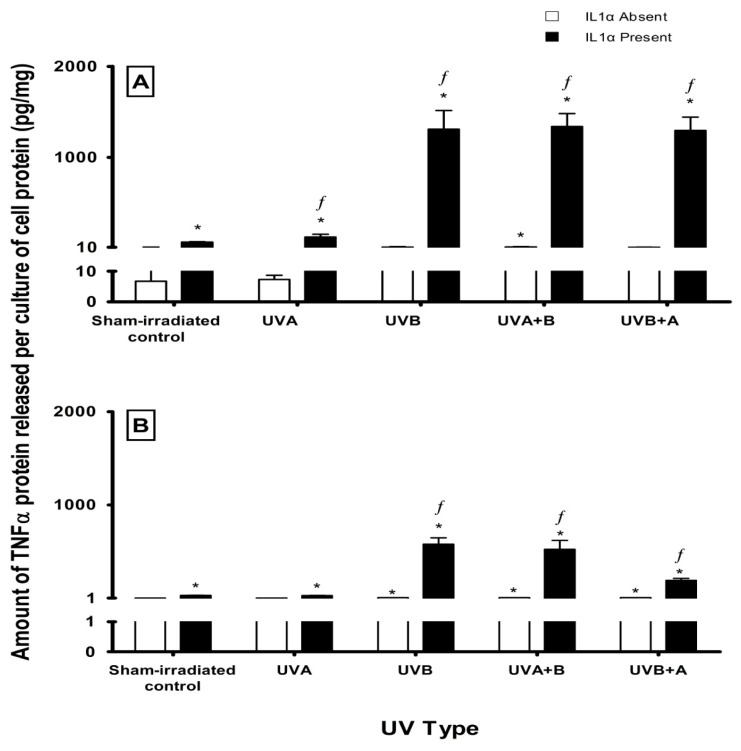
Effect of UV radiation and IL1α on the release of TNFα in (**A**) HEM and (**B**) MM96L cells. Cell cultures were irradiated with the equivalent of 1 MED radiation and treated in the presence or absence of 10 ng/mL IL1α. The media was collected 24 h post-irradiation and assayed for TNFα. Results expressed as the means ± SD from three independent experiments. Statistical analysis was performed using a Student’s paired *t*-test where significance was recorded as *p* ≤ 0.05. (*) Significant difference between Control and UV-irradiated samples. (ƒ) Significant difference between Control and UV-irradiated samples treated with IL1α.

Sham-irradiated HEM cells secreted very low levels of TNFα (7 ± 3 pg/mg cell protein) and these levels only increased slightly (~10 pg/mg cell protein) following UV radiation (Figure 5A). When IL1α was added immediately after UVA-irradiation, TNFα levels increased by 2-fold (control + IL1α: 67 ± 4 pg/mg cell protein, UVA + IL1α: 122 ± 31 pg/mg cell protein). There was a dramatic increase in these levels when IL1α-treated HEM cells were exposed to UVB (1309 ± 206 pg/mg cell protein), UVA + B (1339 ± 142 pg/mg cell protein) and UVB + A (1296 ± 147 pg/mg cell protein) radiation (Figure 5A). In MM96L cells, low levels of TNFα were secreted from both sham- (2 ± 0.2 pg/mg cell protein) and UV-irradiated cells (~5 pg/mg cell protein) (Figure 5B). The addition of IL1α increased TNFα levels to ~30 pg/mg cell protein in the sham- or UVA-irradiated cells. After UVB and UVA + B radiation, IL1α-treated cells secreted 580 ± 69 and 525 ± 95 pg/mg TNFα, respectively while those cells exposed to UVB + A radiation released less TNFα (192 ± 23 pg/mg cell protein) (Figure 5B). Overall, less TNFα was secreted from cells exposed to UVA radiation. The degree by which IL1α increased TNFα secretion from the irradiated cells was greater in HEM cells than compared to MM96L cells (Table 1).

### 2.4. Effect of Pathway Specific Inhibitors on UV-Induced TNFα Release in Melanocyte-Derived Cells

In order to observe which signaling pathway was involved in UV-induced TNFα release, ELISAs were performed to quantify the level of TNFα released from HEM and MM96L cells treated with either a p38 (SB202190), JNK (SP600125) or NFκB (sulfasalazine) inhibitor. Since high dose UVB radiation increased the secretion of TNFα in HEM and MM96L cells (Figure 5), this study was performed using cells only exposed to high dose UVB radiation. The chosen inhibitor doses did not affect the viability of these cells except for sulfasalazine and SP600125, which induced a slight decrease (<10%) in the viability of UVB-irradiated HEM and MM96L cells (results not shown). The cell cultures were treated with the inhibitor for 1 h prior to receiving 1 MED UVB-irradiation (2 kJ/m^2^). After UVB exposure, the cell cultures were incubated with the specific inhibitors for 24 h in the presence or absence of IL1α (10 ng/mL).

**Figure 6:**
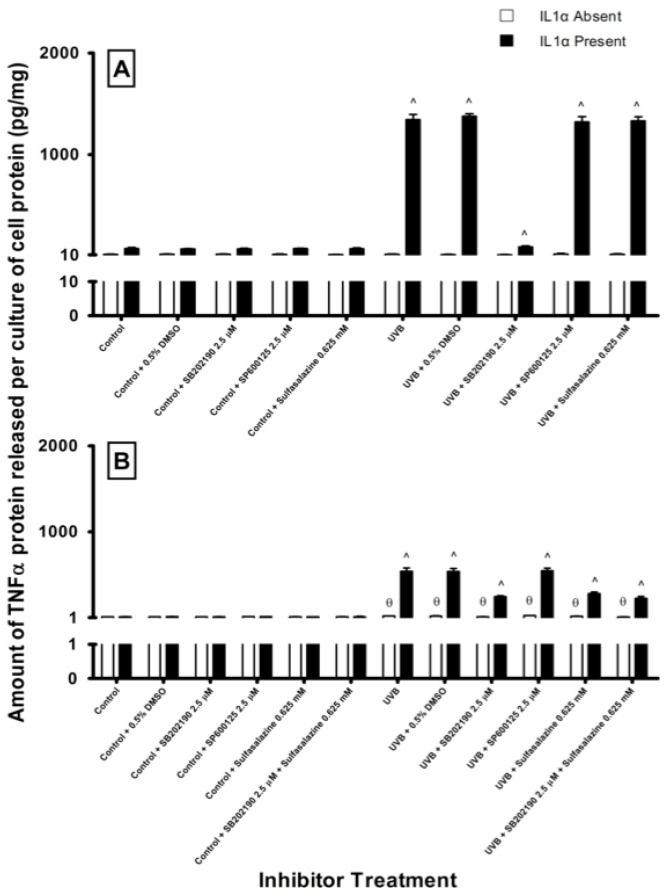
Effect of pathway specific inhibitors on TNFα release from (**A**) HEM and (**B**) MM96L cells at 24 h post UVB-irradiation (2 kJ/m^2^). The cells were incubated with the specific inhibitors for 1 h prior to high dose UVB exposure. After UVB exposure, the cells were incubated for 24 h with the specific inhibitors and treated with or without 10 ng/mL of IL1α. Results expressed as the means ± SD from triplicate samples. Statistical analysis was performed using a Student’s paired *t*-test where significance was recorded as *p* ≤ 0.05. (†)(*) Significant difference between untreated control and inhibitor treated sample in the absence (θ) or presence (^) of IL1α.

In HEM cells, unirradiated cultures treated with either the signaling inhibitors secreted similar amounts of TNFα compared to untreated controls (Figure 6A). SB202190 (2.5 μM) inhibited the release of TNFα (31% inhibition) from UVB-irradiated cells compared to their untreated irradiated cohorts (16 ± 4 pg/mg cell protein). This inhibition was more pronounced when these UVB-irradiated cells were stimulated with IL1α. The irradiated cells secreted 1343 ± 51 pg/mg TNFα after 24 h, however when these cultures were treated with SB202190, TNFα secretion was inhibited by 94%. Neither SP600125 nor sulfasalazine inhibited TNFα secretion from the UVB-irradiated HEM cells either in the presence or absence of IL1α.

In MM96L cells, pathway specific inhibitors had no effect on TNFα secretion in sham-irradiated cells (Figure 6B). UVB-irradiated MM96L cells secreted 20 ± 1 pg/mg TNFα, and this fell by 55% when these cells were treated with 2.5 μM SB202190. UVB-irradiated MM96L cells incubated in the presence of IL1α, secreted 539 ± 40 pg/mg TNFα over 24 h. The addition of SB202190 to these cells caused a significant reduction in the secretion of TNFα (55% inhibition). SP600125 did not inhibit the release of TNFα from UVB-irradiated MM96L cells either in the presence or absence of IL1α (Figure 6B).

0.625 mM Sulfasalazine slightly inhibited TNFα release from UVB-irradiated MM96L cells by 25% when compared to their untreated irradiated cohort (20 pg/mg cell protein). When the irradiated cells were treated with IL1α, sulfasalazine inhibited TNFα secretion by 48% compared to uninhibited cells treated with IL1α (539 ± 40 pg/mg cell protein). There was an additive effect observed when SB202190 and sulfasalazine were both added to the irradiated cells treated with IL1α, where TNFα levels fell by 58%. The results suggest that the p38 MAPK pathway is involved in UVB-mediated TNFα release in both cell types.

**Figure 7:**
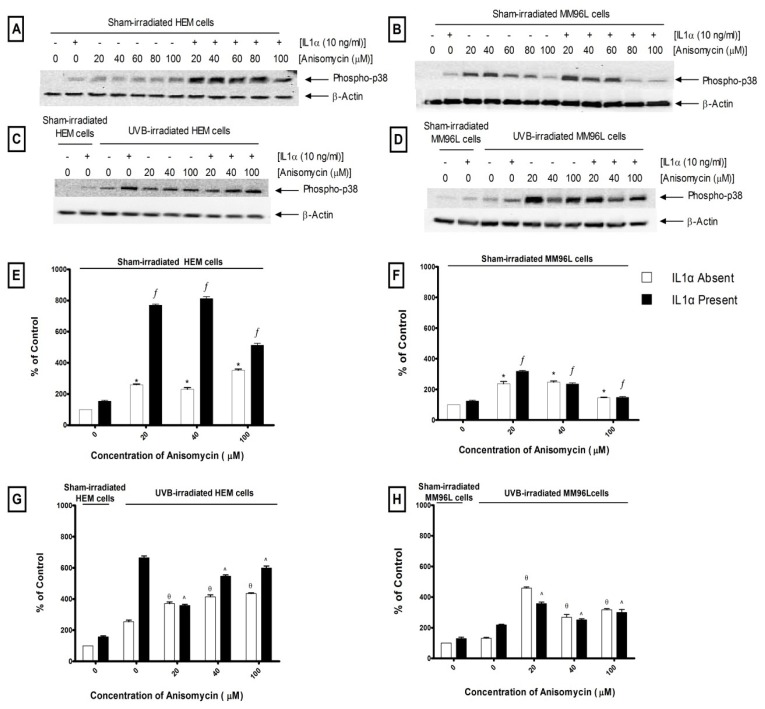
The effect of anisomycin on the expression of phospho-p38 protein in HEM and MM96L cells. Representative western blots probed for phospho-p38 in (**A**,**C**) HEM and (**B**,**D**) MM96L cells treated with anisomycin. (**E**,**F**) Sham and (**G**,**H**) UVB-irradiated (2 kJ/m^2^) cell cultures treated with or without 10 ng/mL of IL1α were incubated with anisomycin for 15 min. Results expressed as the means ± SD from triplicate samples. Statistical analysis was performed using a Student’s paired *t*-test where significance was recorded as *p* ≤ 0.05. Significant difference between untreated control and anisomycin treated sample in the (*) absence or (ƒ) presence of IL1α. Significant difference between UVB-irradiated untreated sample and anisomycin treated irradiated sample in the (θ) absence or (^) presence of IL1α.

Anisomycin, a known activator of the p38 MAPK pathway, was used to confirm if the p38 MAPK-mediated TNFα release is a UV specific response (Figure 7A) [31]. There was a dose-dependent decrease in the viability of attached cells treated with anisomycin (20–100 μM) in sham- or UVB-irradiated HEM (80%–25%) or MM96L cells (60%–7%) (results not shown). In sham-irradiated HEM cells, anisomycin (20–100 μM) increased phospho-p38 levels by 3- to 4-fold compared to that of untreated controls (Figure 7B). When IL1α was added to these cultures, there was a further increase (5- to 8-fold) of phospho-p38 levels compared to that seen in the untreated control. In UVB-irradiated HEM cells, there was a 3-fold increase in phospho-p38 levels compared to that seen in sham-irradiated controls (Figure 7D). When anisomycin (20–100 μM) was added to UVB-irradiated cells, it resulted in a ~2-fold increase in phospho-p38 levels. A similar result was also seen when anisomycin was added to UVB-irradiated cells treated with IL1α (Figure 7D).

In MM96L cells, low dose anisomycin (20 μM) increased phospho-p38 levels by 2-fold compared to that of untreated controls (Figure 7C). However, increasing anisomycin concentrations resulted in a drop in phospho-p38 levels (Figure 7C). When IL1α was added to the unirradiated cells, phospho-p38 levels were only higher in those cells treated with 20 μM anisomycin (Figure 7C). In UVB-irradiated MM96L cells, anisomycin (20–100 μM) increased phospho-p38 levels by 2- to 4-fold compared to that seen in the untreated irradiated cells (Figure 7E). Phospho-p38 levels fell when IL1α was added to anisomycin treated irradiated cells (Figure 7E). The results show that anisomycin activated the p38 pathway in HEM and MM96L cells.

**Figure 8:**
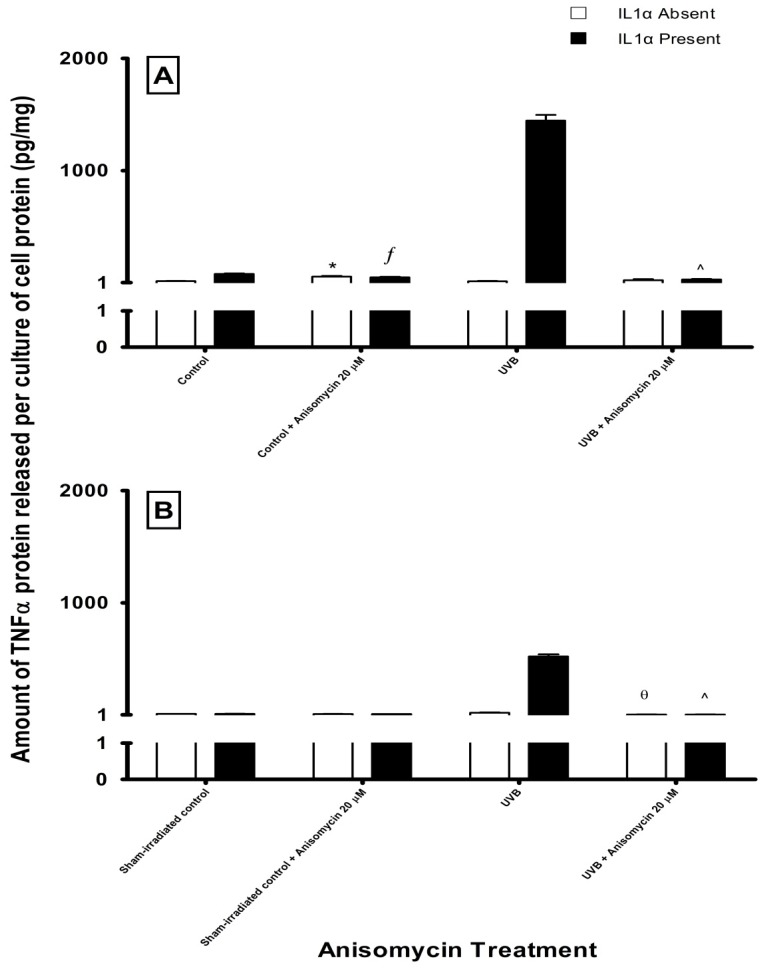
The effect of anisomycin on TNFα release in (**A**) HEM and (**B**) MM96L cells at 24 h post-treatment. Cell cultures were treated with either 20 or 40 μM anisomycin in sham-irradiated or UVB-irradiated (2 kJ/m^2^) cells in the presence or absence of 10 ng/mL of IL1α. Results expressed as the means ± SD from triplicate samples. Statistical analysis was performed using a Student’s paired *t*-test where significance was recorded as *p* ≤ 0.05. Significant difference between untreated control and anisomycin treated sample in the (*) absence or (ƒ) presence of IL1α. Significant difference between UVB-irradiated untreated sample and anisomycin treated UV-irradiated samples in the (θ) absence or (^) presence of IL1α.

When anisomycin was added to sham- or UVB-irradiated HEM and MM96L cells, almost no increase in TNFα secretion was observed (Figure 8). A similar result was also observed for cells treated with IL1α. Even though anisomycin did activate the phosphorylation of p38 MAPK (Figure 7), it did not enhance TNFα release from UVB-irradiated HEM and MM96L cells in the presence or absence of IL1α, which suggests that the downstream events activated by UV radiation differ to that of anisomycin. However, in UVB-irradiated cells treated with IL1α, anisomycin addition resulted in a 98% reduction in TNFα released from both HEM and MM96L cells.

## 3. Discussion

### 3.1. Choice of Cell Types and UV Radiation

Two different human melanocyte-derived cell types, HEM (Human Epidermal Melanocytes) and MM96L (Malignant Melanoma) cells were used in this study. The MM96L cells have acquired a B-Raf V600E mutation and do not express the p16 protein but they do possess functional N-Ras and PTEN [32,33]. These cells were cultured from a secondary tumor of a 66 year old female melanoma patient, however the location of which was not stated [34]. HEM and MM96L cells were used to observe how normal and cancerous melanocytes responded to UV radiation. These cells were exposed to a low and high dose of UVA, UVB, UVA + B and UVB + A radiation. The dose used in this study was that UV component found in 1 MED sunlight (40–50 kJ/m^2^) where a high dose UVA was 40 kJ/m^2^ and UVB was 2 kJ/m^2^, while the low doses used corresponded to the UV components seen in 0.1 MED [22].

HEM cells were less susceptible to different UV types or doses than were MM96L cells (Figure 1), and this suggests that these cells either have a more efficient DNA repair mechanism or certain factor(s) rendering them less susceptible to UV radiation. This factor is most likely to be melanin, which is a UV-absorbing pigment synthesized in melanocytes [35,36]. Kobayashi *et al.* [37] observed that melanin forms supranuclear caps around the nucleus protecting the DNA from harmful UV radiation [37]. As such, although melanocytes possess a weaker antioxidant defence mechanism than do keratinocytes, they are less sensitive to UV-induced damage [38].

The highest levels of cell death were observed in cells exposed to high dose UVB, UVA + B and UVB + A radiation. Since UVA alone inflicted less damage to the cells, the UVB component is predominant over UVA in the combination of UVA + B or UVB+A radiation used (Figure 1B). Koch-Paiz *et al.* [39] found that UVA (50 kJ/m^2^) triggered a weaker genetic response than UVB (0.1 kJ/m^2^) radiation in MCF-7 cells. In mouse embryonic fibroblast cells, UVA-induced (180 kJ/m^2^) DNA damage was repaired within 30 min as opposed to that of UVB (0.8 kJ/m^2^), which were repaired after 24 h [40]. This suggests that UVB radiation induces greater damage and cell death than does UVA radiation. In addition, Schieke *et al.* [41] found that keratinocytes responded differently to UVA and UVB radiation alone but when a combination of radiation was used (UVA + B or UVB + A), a “third” response was created that resembles neither UVA nor UVB alone.

### 3.2. Effect of UV Radiation on the Activation of the p38 MAPK, JNK and NFκB Pathways in Melanocyte-Derived Cells

In response to UV radiation, the p38 MAPK and JNK pathways were activated differently in HEM and MM96L cells suggesting that the UV response is a cell type-dependent effect (Figures 2 and 3). In the HEM cells, although there were differences in phospho-p38 levels, the pathway was activated in a similar pattern irrespective of the UV types and doses used (Figure 2B,D). In MM96L cells, minimal activation of phospho-p38 MAPK was seen in cells exposed to low dose UV radiation irrespective of the type used (Figure 2C). In these cells, high dose UVA and UVA + B radiation induced a dramatic increase in p38 MAPK activation while that following UVB and UVB + A radiation was low and transient (Figure 2E). In MM96L cells, this pathway appeared to be both UV wavelength and dose-dependent. In further support of this proposal, Liu *et al.* [3], found that the transcription factor MiTF was degraded in melanocytes and melanoma cells following exposure to UVA but not UVB radiation, which shows that the signaling pathways activated by both types of UV differ in these cells.

Both HEM and MM96L cells predominantly expressed higher levels of JNK1 compared to JNK2 (Figure 3). This suggests that the JNK1 isoform is selectively activated in response to UV-induced stress. Some melanoma cells (1205Lu, WM983B, sk28, WM852 and WM 793) possess a high JNK1/JNK2 ratio while others (888mel, Gerlach and WM983A) have a low ratio [8]. The exact role played by each JNK isoform in melanoma is unclear. It was shown that JNK1 siRNA inhibited cell growth in melanoma cell lines expressing high levels of JNK1 whereas JNK2 siRNA had no effect [8]. However, in WM983B melanoma cells JNK inhibition did not affect cell growth but induced apoptosis [8].

Higher levels of phospho-JNK2 were observed in UV-irradiated MM96L cells but not in HEM cells where it was almost negligible (Figure 3). In experiments involving JNK2-deficient fibroblasts, this JNK isoform was shown to be a negative regulator of cell proliferation [42]. As JNK2 is expressed in MM96L cells, its postulated role in apoptosis could have contributed to the sensitivity of these cells to UV-induced cell death (Figure 1). However, *in vivo* studies using JNK2 knockout mice showed that TPA-induced tumor growth was inhibited, which suggest that it is necessary for tumor proliferation [43]. Tao *et al.* [44] found that mice containing JNK2^−/−^ CD8^+^T cells exhibited resistance to tumor proliferation and development when inoculated with B16F0 melanoma cells. This suggests that JNK2 signaling is involved in cell proliferation or cell death.

The ERK pathway has been suggested to be the main pathway involved in melanoma formation and progression [2,3,45–47]. Less is known about the role played by p38 MAPK and JNK pathways in this process. Ras and Raf are also located upstream of the p38 MAPK and JNK pathways and any mutations present in these upstream proteins will affect the ERK1/2, p38 MAPK and JNK pathways. Since MM96L cells possess B-Raf mutation [48], it may explain the higher levels of p38 and JNK in these cells post-high dose UV radiation. Estrada *et al.* [10] have shown that both high levels of ERK and p38 MAPK activity are required for melanoma development. These authors also found that inhibition of p38 MAPK activity alone can inhibit migration of melanoma cells [10]. The JNK pathway can be activated by ERK in a feedback loop and both pathways can activate cyclin D1 which is a positive regulator of cell cycle progression in melanoma cells [49]. Therefore, further research into the involvement of p38 MAPK and JNK, aside from the ERK, in melanoma formation is warranted.

The NFκB pathway was minimally activated in both HEM and MM96L cells in response to different UV types and doses (Figure 4). MM96L cells possessed slightly higher levels of constitutively active phospho-IκBα than did HEM cells. McNulty *et al.* [16] also found that melanocytes and melanoma cells express high constitutive levels of NFκB that were not augmented by UVB-irradiation. UVA radiation has been shown to have no effect on NFκB levels and activity in normal melanocytes [50]. These results suggest that UV radiation may not activate the NFκB pathway to the same extent as seen in the p38 MAPK and JNK pathways. It is also possible that together with the constitutive activation of NFκB, the high activation of p38 and JNK pathways post-UV radiation may not stimulate further NFκB activation following UV radiation.

### 3.3. Effect of UV Radiation and IL1α on TNFα Release in Melanocyte-Derived Cells

The level of TNFα released from both cell lines was less following UVA radiation compared to UVB radiation (Figure 5). This difference could be due to the ability of UVA radiation to selectively regulate cytokines, promote protein degradation or inhibit protein synthesis [51,52]. UVA upregulated the transcription of IL12 but at the same time inhibited that of IL10 suggesting that it may also selectively downregulate TNFα transcription in melanocyte-derived cells [30,52]. In these cells, UVA + B radiation caused a similar increase in TNFα levels to that of UVB radiation. UVB + A radiation on the other hand, decreased TNFα levels in MM96L cells but not in HEM cells (Figure 5). As UVA radiation induced less TNFα secretion than UVB, it is possible that in the combination of UVB + A radiation, the UVA component may have a suppressive effect on the release of this cytokine in MM96L cells. In HEM cells this suppressive effect brought about by UVA may have been overshadowed by the molecular interaction induced by UVB which suggests that there is a cell type-dependent response to UV radiation (Figure 5). MM96L cells do not contain large quantities of intracellular TNFα as determined by ELISA using cell lysates (results not shown). This suggests that the reduced level of TNFα secreted from these cells is most likely due to reduced synthesis. When IL1α was added to the cells, a greater increase in TNFα release was observed in the UV-irradiated HEM cells compared to MM96L cells (Figure 5). TNFα has been shown to have a pro-survival effect in different cell lines although anti-survival effects have also been reported [11,18,53,54]. Ivanov *et al.* [18] found that ATF2 downregulated TNFα expression in UVC-irradiated (0.06 kJ/m^2^) melanoma cells. Forced expression of ATF2 increased UVC-induced cell death in melanoma cells while the addition of exogenous TNFα restored cell survival. In this study, HEM cells were less sensitive to UV radiation than MM96L cells and it is possible that high levels of TNFα could have protected these cells from UV-induced cell death while the lower levels in MM96L could have made them more susceptible (Figure 1).

The addition of IL1α to both cell lines in general enhanced p38 and JNK activity but not NFκB (results not shown). In order to elucidate which of these signaling pathway(s) were involved in UV + IL1α-induced TNFα secretion, specific inhibitors were used. Inhibition of the p38 MAPK but not that of JNK or NFκB pathway significantly reduced TNFα levels in UVB-irradiated HEM cells (Figure 6A). In the UVB-irradiated MM96L cells, inhibition of either the p38 MAPK or NFκB pathways caused a partial decrease in TNFα levels (Figure 6B). These results suggest that the p38 MAPK pathway is the main pathway involved in regulating UV-induced release of TNFα from melanocyte-derived cells. Ivanov *et al.* [11] found that inhibition of p38 MAPK pathway led to a decrease in TNFα transcriptional activation. Since both p38 MAPK and NFκB inhibitors partially decreased the secretion of TNFα from MM96L cells, crosstalk may exist between these and other pathways as the p38 MAPK pathway was shown to be upstream of the NFκB pathway in A2058 melanoma cells [55].

In order to confirm the inhibitory effect of SB202190, anisomycin, a potent stimulator of the p38 MAPK and JNK pathways was used (Figure 7) [31]. While anisomycin increased phospho-p38 MAPK activity, it was unable to stimulate TNFα release in the presence or absence of IL1α in sham-irradiated cells (Figure 8). This suggests that p38 MAPK-mediated TNFα release following UVB-irradiation may be a UV-specific response. However, when anisomycin was added to UVB-irradiated cells, TNFα release was not observed even if IL1α was present (Figure 8). It is possible that UVB and anisomycin activate the p38 MAPK pathway via a different mechanism and the mode of activation might change when both stimuli are used together. In support, Ravi *et al.* [55] found that while caffeine or rottlerin inhibited UV-induced p38 MAPK activation neither was able to inhibit anisomycin-induced p38 MAPK activation. This suggests that UV radiation has different upstream/downstream intermediates to that of anisomycin.

In summary, HEM and MM96L exhibit different responses to UV radiation. The MAPK pathways are involved in a plethora of functions like proliferation, inflammation, apoptosis, differentiation, and cell cycle regulation among others [56,57]. As the p38 MAPK and JNK pathways are regulated differently in HEM and MM96L cells, it suggests that the functions performed by both in melanocytes may not be the same as in melanoma cells. While these pathways are usually involved in maintaining homeostasis in normal cells they may be involved in pro-tumorigenic activities in compromised cells. This may in part be due to the B-Raf mutation and other mutations acquired in MM96L cells which is upstream of the MAPK pathways. These pathways may act on their own or in conjunction with ERK to promote oncogenesis. As such, besides the ERK pathway, the p38 and JNK pathway should be probed further in identifying their supportive roles in melanomagenesis.

TNFα secreted following irradiation is a UV specific response and the p38 MAPK pathway appears to be the main pathway involved in HEM cells but not MM96L cells, however the NFκB pathway may also be involved. Currently, the exact role played by TNFα in skin carcinogenesis is not known. Studies have shown that TNFα has a role in executing either pro- or anti-tumor activities [11,18,20]. Despite its dual role, identifying the pathways regulating UV-induced TNFα release is of importance because if it is an anti-tumor agent then pharmacological enhancers of the p38 MAPK pathway may increase its expression in targeted tumors. On the other hand, if it has a pro-tumor function, pharmacological inhibitors of the p38 MAPK pathway could be useful in reducing TNFα levels to eradicate skin tumors. Since the p38 MAPK pathway is also involved in normal homeostasis, the challenge would be to intervene in p38 MAPK-mediated TNFα release without initiating any instability within the cell by disrupting other roles of this pathway, which are responsible for normal cellular functioning. We are currently undertaking further studies on the role p38 MAPK plays in regulating TNFα release in UV-irradiated melanocyte-derived cells.

## 4. Experimental Section

### 4.1. Materials

All tissue culture media and supplements were obtained from Invitrogen (Melbourne, Australia) except for FBS (Foetal Bovine Serum) and BSA (Bovine Serum Albumin), which were obtained from Bovogen (Melbourne, Australia). SB203580 (p38 MAPK inhibitor), SP600125 (JNK inhibitor), NFκB Inhibitor II were from Merck (Melbourne, Australia). The chemilucent kit, Goat-HRP conjugated anti-rabbit immunoglobulin and anti-mouse immunoglobulin were obtained from Millipore (Sydney, Australia). The primary antibodies (phospho-p38 rabbit polyclonal antibody, phospho-JNK rabbit polyclonal antibody, phospho-IκBα mouse monoclonal antibody, β-actin) were from Genesearch (Gold Coast, Australia) and AccuKine Human TNFα ELISA Kit was from Scientifix (Melbourne, Australia). All other chemicals were obtained from Sigma (Sydney, Australia), unless otherwise indicated. All tissue culture vessels were obtained DKSH (Melbourne, Australia), while the Microcon YM-10 micro-concentrators were from Millipore (Sydney, Australia).

### 4.2. Cell Types

The HEM (Human Epidermal Melanocytes) cells obtained from Banksia Scientific (Brisbane, Australia) and MM96L melanoma cells [34] were kindly donated by Dr Glen Boyle (QIMR, Brisbane, Australia) were grown in culture at 37 °C. HEM cells were cultured with Medium 254 supplemented with 1% (*v*/*v*) Human Melanocyte Growth Supplement and 1% (*v*/*v*) Penicillin-Streptomycin-Glutamine (10,000 units/mL penicillin G sodium, 10,000 μg/mL streptomycin sulfate and 29.2 mg/mL l-glutamine). The spent culture media was discarded and replaced with fresh media every two to three days. MM96L cells were cultured with RPMI medium 1640 supplemented with 5% (*v*/*v*) FBS and 1% (*v*/*v*) Penicillin-Streptomycin-Glutamine. Spent culture media was removed and discarded every three to four days and replaced with fresh RPMI media.

#### Subculture

When the HEM and MM96L cell cultures reached confluence, the respective spent culture media were aspirated and the cells washed twice with sterile phosphate-buffered saline (PBS) and once with Trypsin-EDTA solution. After which, the cells were incubated with sterile Trypsin-EDTA solution and the trypsinized cells were used to seed the petri dishes or 6-well plates used in experiments. All solutions used in tissue culture were kept at 37 °C for MM96L cells and at RT (20 °C) for HEM cells unless specified otherwise.

### 4.3. UV-Irradiation

The UV cabinet (Wayne Electronics, Sydney, Australia) housed 6 UV fluorescent lamps: 3 UVA Phillips Ultraviolet TLK 40W/10 R lamps (Phillips, Eindhoven, Holland) and 3 UVB Phillips Ultraviolet TL 20W/01 RS lamps (Phillips, Eindhoven, Holland). The UV light emitted from UVA lamps ranged from 350 to 400 nm with a peak at 365 nm while that of UVB lamps (305–315 nm) had a maximal output at 311–312 nm [58,59]. The variation in the output (mW/cm^2^) of the UV lamps was measured using a relevant UV detector (UVA or UVB) attached to an IL-1400A Photometer (International Light, Newburyport, USA). The low and high doses used in this study represented the respective UV (A and B) components of either 0.1 or 1 MED [22]. Kuchel *et al.* [60] found that the average UV dose to induce 1 MED was 41 ± 2 kJ/m^2^ when exposed to solar simulated UV light. As it is estimated that the UVA and UVB component of sunlight is 95% and 5% respectively, 40 kJ/m^2^ of UVA and 2 kJ/m^2^ of UVB was chosen to represent 1 MED of solar sunlight (40 kJ/m^2^) while a tenth of these doses (UVA: 4 kJ/m^2^ and UVB: 0.2 kJ/m^2^) represented 0.1 MED. When the cells were exposed to both UVA and UVB radiation, as it was not possible to simultaneously irradiate with both UV types they were exposed initially first to either UVA (denoted UVA + B) or UVB (denoted UVB + A). The irradiation protocol was performed as described by Huynh *et al.* [61].

### 4.4. Cell Viability

Cell viability was determined 24 h post-UV radiation using the Trypan Blue exclusion method [61].

### 4.5. Inhibitor and Anisomycin Studies

All the signaling pathway inhibitors and anisomycin were dissolved in DMSO. In the inhibitor studies, cells cultured in 60 mm petri dishes were pre-treated for 1 h with either 2.5 μM SB203580 (p38 MAPK inhibitor, Stock solution: 2 mM), 2.5 μM SP600125 (JNK inhibitor, Stock solution: 2 mM) or 0.625 mM sulfasalazine (NFκB inhibitor, Stock solution: 0.25 M). After which the inhibitor solution was removed and the cell culture was irradiated. Immediately following radiation, the inhibitor solution was added to the culture and was incubated for various time points as seen in the results section. In the anisomycin studies, irradiated cell cultures were incubated with 20–100 μM anisomycin (Stock solution: 20 mM) for various time points as seen in the results section.

### 4.6. Western Blotting

The cells were harvested 0–120 min post-irradiation as previously described [17,61]. The nylon membranes were incubated with the relevant antibody [1:1000 phospho-p38 rabbit polyclonal antibody, 1:1000 phospho-JNK rabbit polyclonal antibody, 1:1000 phospho-IκBα mouse monoclonal antibody, 1:1000 β-actin (loading control)] overnight at 4 °C. After which, they were incubated with the appropriate secondary antibody (1:1000 Goat-HRP conjugated anti-rabbit immunoglobin or 1:1000 Sheep HRP conjugated anti-mouse immunoglobin). The membranes were developed in Chemilucent solution and visualized using a Chemidox XRS unit (BioRad). The digital image was analyzed for densitometry using Quantity One Digital Imaging Software Version 4.5.1 (BioRad). The intensity of the expression of the phosphorylated signaling intermediate was first normalized to that of its loading control β-actin, and for the sham-irradiated untreated controls this was expressed as 100%. The value for the phosphorylated signaling intermediate at each time point was compared to that of the sham-irradiated controls and was expressed as a percentage.

### 4.7. ELISA

The levels of TNFα released from the UV-irradiated cell cultures were measured 24 h post-irradiation. Immediately after UV exposure, fresh media was added to the cells. In some experiments, 10 ng/mL of IL1α was added to the media as it stimulates TNFα release from UV-irradiated keratinocytes [30]. Aliquots of the culture media were concentrated using Microcon YM-10 micro-concentrators. The levels of TNFα in these media samples were determined using an AccuKine Human TNFα ELISA Kit.

### 4.8. Statistical Analysis

The results obtained in this study were expressed as the mean ± standard deviation (SD) from triplicate samples. The statistical significance was determined by the use of Student’s paired, one-tailed *t*-test with *p* ≤ 0.05 deemed to be significant.

## 5. Conclusions

We have found that the MAPK pathways are activated differently in normal and compromised melanocytes. Furthermore, we have found that the p38 pathway plays a major role in the secretion of an inflammatory molecule, TNFα in UV-irradiated melanocyte-derived cells but not via anisomycin-activated p38 MAPK pathway. Therefore, further investigation into the signaling intermediates regulating TNFα secretion in UV-irradiated melanocytes and melanoma cells is warranted.

## Figures and Tables

**Table 1:** Effect of IL1α on the release of TNFα from UV-irradiated melanocyte-derived cell line.

Cell Line	Sham	UVA	UVB	UVA + B	UVB + A
HEM	10 ± 4	17 ± 6	109 ± 40	103 ± 26	130 ± 24
MM96L	15 ± 1	14 ± 4	97 ± 22	88 ± 23	32 ± 4

All values are calculated as the fold-increase ± SD of UV-irradiated cells treated with IL1α compared to their corresponding irradiated counterparts. Values calculated from the data represented in Figure 5.
